# Botulinum Toxin Type A in the Treatment of Raynaud’s Syndrome: A Case Report

**DOI:** 10.7759/cureus.57327

**Published:** 2024-03-31

**Authors:** Plamen Penchev, Valentin Dobrev, Petar-Preslav Petrov, Remzi Hyusein, Vladislav Velchev, Kristiyan Georgiev

**Affiliations:** 1 Faculty of Medicine, Medical University of Plovdiv, Plovdiv, BGR; 2 Department of Neurology, Medical Center "VIP", Targovishte, BGR; 3 Department of Anatomy, Histology and Embryology, Medical University of Plovdiv, Plovdiv, BGR; 4 Faculty of Medicine, Medical University of Sofia, Sofia, BGR; 5 Clinic of Neurology, University Multiprofile Hospital for Active Treatment (UMHAT) Saint Marina, Varna, BGR; 6 Department of Neurology and Neuroscience, Medical University of Varna, Varna, BGR

**Keywords:** xeomin, case report, botulinum toxin, botulinum toxin type a, raynaud’s syndrome

## Abstract

Raynaud's syndrome is characterized by paroxysmal vasospasm in the digital arterioles, following exposure to cold or stress. Pain, swelling, stiffness, and hypoesthesia are observed as manifestations. The presence of a trophic ulcer is accompanied by a range of severe manifestations. The assaults occur in three distinct phases, namely vasospastic, plethoric, and erythema. Various approaches improve the overall well-being of a patient. It is possible to differentiate between primary and secondary Raynaud's syndrome, the latter being linked to systemic diseases. The application of botulin toxin is commonly indicated in several medical conditions including focal dystonia, spasticity with or without contractures, paraparesis in children with cerebral palsy, multiple sclerosis, brain injuries, involuntary muscle hyperactivity of a non-dystonic nature, pain management, strabismus, nystagmus, sialorrhea, and esthetic medicine. When treating Raynaud’s a technique is used with injection at the base of each finger, from the palmar side, which helps with cooling and minimizing discomfort for patients. We present a clinical case of a 70-year-old female patient with Raynaud’s syndrome in which we have placed 70E distributed to both hands botulin toxin type A. Improvement in the patient’s symptomatology was noticed on day 3, with warming of the hands, lack of swelling, and pain with duration of the effect little over three months. The patient underwent a six-month follow-up following the therapy with botulinum toxin type A, and no indications of recurrence or advancement of Raynaud's syndrome (RS) were seen.

## Introduction

Raynaud's phenomenon (RP) is a condition characterized by the constriction of blood vessels in the digital vessels, which is caused by exposure to cold or stress. The condition is predominantly detected in the hands, although it frequently manifests in the toes [1]. The standard characterization of transitory digital ischemia involves three distinct phases: (1) ischemia, (2) cyanosis, and (3) reperfusion. During these phases, patients may exhibit color changes ranging from white to blue to red [2]. The incidence of RP in the whole population ranges from 3% to 5%, with higher occurrence observed in women, with a female-to-male ratio of 4:1 [1,2]. Severe pain, paresthesia, and swelling of the fingers and toes are frequently reported by patients.

The primary cause of RP (Raynaud's disease) is unknown. Secondary RP (Raynaud's syndrome) can be attributed to several factors and frequently arises alongside underlying autoimmune or connective tissue disorders. Scleroderma is the predominant etiology, with Raynaud's syndrome (RS) affecting 90% of individuals. Additional factors contributing to this condition encompass Sjogren's illness, rheumatoid arthritis, systemic lupus erythematosus (SLE), the efficacy of the contraceptive pill/oral contraceptives, and β-blockers.

These individuals exhibit delayed presentation, characterized by heightened disease severity and the presence of accompanying comorbidities, including ulceration, gangrene, and even amputation [3]. The therapeutic approach holds significant importance. The medicinal interventions for RP are contingent upon the root cause and extent of the condition. The treatment of patients with secondary Raynaud's syndrome (RP) presents more therapeutic challenges due to the potential exacerbation of digital vasospasm by concurrent disease symptoms. The neurotoxin known as botulinum toxin A (Btx-A) is synthesized by the bacterium, *Clostridium botulinum* [4].

The aim of this case report is to illustrate the effectiveness of botulinum toxin type A in the management of RS in a female patient aged 70 years. This study offers valuable insights into the treatment outcomes, dosage adjustments, and administration techniques associated with botulinum toxin A, resulting in enhancing our comprehension of its potential as an effective treatment for RS.

## Case presentation

**Figure 1:**
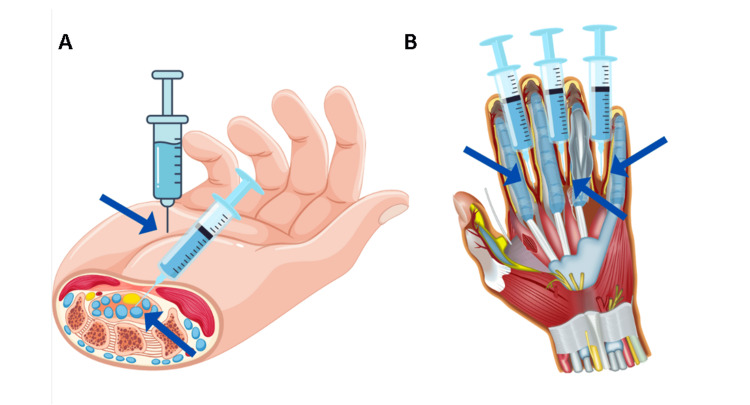
Botulinum toxin administration technique A) Injection technique at the palmar side of the hand (arrow). B) Injection technique at the base of the fingers (arrows). Image credits: Made by the author Plamen Penchev with Canva.

It is recommended that the patient provide informed consent for the administration of medication containing botulinum toxin type A. Those drugs are not officially registered for the treatment of RS. Guidelines for diluting the drug differ between the different molecules used. If possible, the smallest dilution is used, as larger dilutions cause significant pain in the hand [5]. Prior to the instillation of the toxin, the skin is topically cleaned with a disinfectant that does not contain alcohol. Afterward, local anesthesia with ice is used, followed by the administration of an injection using a 30-33G needle. Every hand receives 50E, distributed across the interdigital space at the base of each finger, specifically from the palmar side, which helps with cooling and minimizes discomfort for patients (Figures 1A, 1B).

**Figure 2:**
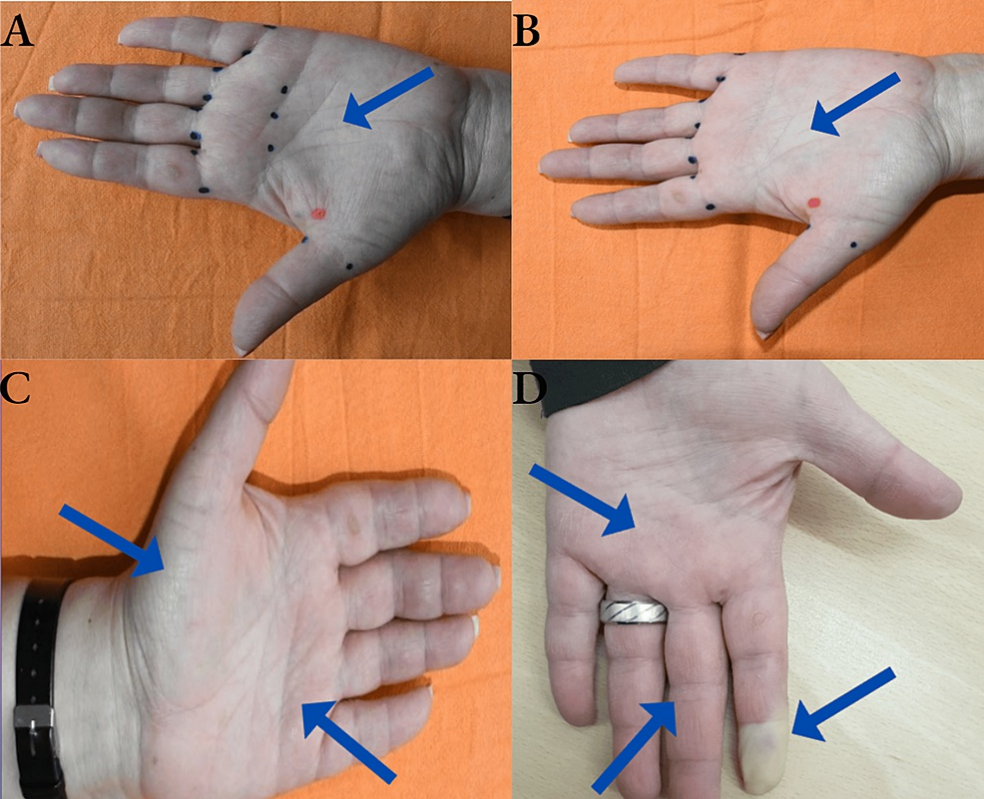
Results after the administration of botulinum toxin A A) Prior to the procedure, and the arrow shows the symptoms of the patients. B) The first improvement is seen on the third day with warming of the zones, and the arrow shows improvement of the symptoms as the hand gets warmed. C) Increased warming of the hands, without swelling or pain, and the arrow on the white area shows still not warmed enough area. D) Symptom relapse at the end of the six-month follow-up. The one arrow that shows the white part is the symptoms relapse the most and the other arrows are symptom relapse slower.

We present a clinical case of a 70-year-old female patient with Raynaud’s syndrome. Her diagnosis was established at least 20 years prior, during this time she was treated with oral medications, beta-blockers, calcium antagonists, etc., with unsatisfactory and short-term effects. The patient was informed about the possible treatment option with botulinum toxin type A and gave her consent to the treatment. On the day of injection, she had clear discoloration of the distal phalanges, with paresthesia and hyperpathy of the discolored areas; temperature asymmetry was also present. We administered a total of 70E, distributed to both hands: 35E each was distributed across the marked injection sites and botulinum toxin type A was applied at the base of the fingers, specifically from the palmar side (Figure 2A). Improvement in the patient’s symptomatology was noticed on day 3 (Figure 2B), with warming of the hands (Figure 2C) without swelling and pain with duration of the effect over three months. The patient underwent a six-month follow-up following the therapy with botulinum toxin type A, and no indications of recurrence or advancement of RS were seen. At the end of the sixth month, there was some relapse of the symptoms with discoloration, paresthesia, and hyperpathy on the distal phalanx of the index finger (marked in Figure 2D).

## Discussion

The most prevalent ischemia condition encountered by hand surgeons is the Raynaud phenomenon (RP) [6]. The estimated prevalence ranges from 3% to 21%, with females being 10 times more likely than males [2-4]. These prevalences are confirmed as the patient in our case is also female, so we have a correlation with the previously cited studies. The etiology of ischemia remains not well established; however, it is theorized that this condition arises due to an imbalance between vasodilatory and vasoconstrictive components [1,3,5]. The individual's quality of life can be significantly compromised by severe ischemia of the digits caused by RP, leading to discomfort, ulceration, loss of fingers/toes, and disability.

Iorio et al. report that the criteria for primary Raynaud's syndrome include peripheral vasospasm episodes caused by cold or stress, symmetrical engagement of both hands, absence of wound necrosis or gangrene, lack of any other underlying medical condition, normal capillaroscopy observation results, normal values of erythrocyte sedimentation speed (ESS), and negative anti-nuclear antibodies test [6]. In our case, the patient experienced episodes of peripheral vasospasm with symmetrical involvement of both hands. These episodes were triggered by exposure to cold and stress. There was no evidence of wound necrosis or gangrene, and there was no underlying pathology. The capillaroscopy results were normal, and the levels of ESS were normal. Additionally, the anti-nuclear antibodies test was negative.

The primary therapeutic interventions for Raynaud's syndrome (RS) include lifestyle adjustments, including the cessation of smoking, avoidance of cold exposure, and the reduction of anxiety and stress levels. Several oral medicines have been employed with varying degrees of efficacy. The effectiveness of the treatment varies, and there is no universally applicable treatment plan [2,5]. Periarterial sympathectomy, first suggested by Flatt in 1980, represents an additional therapeutic approach employed for cases of severe refractory Raynaud's syndrome (RS) [7]. Nevertheless, the technique may be associated with notable postoperative morbidity due to the specific location of the incisions. The technical complexity of future revisions to this operation increases in the event of symptom recurrence [5]. Furthermore, the lack of consistency in technique can result in fluctuations in the results [6,7].

The term "Raynaud phenomenon," which was first used by Maurice Raynaud in 1862, describes the transient vasospasm of arteries brought on by cold or stress, which is followed by painful reactive hyperemia of the digits, blanching, and cyanosis [2]. Our patient exhibited her clinical symptoms during the winter, indicating that the cold is the primary precipitating factor. Additionally, she experienced these symptoms during stressful situations, therefore establishing a correlation with Maurice Raynaud's results. The condition is believed to arise from an overreaction to sympathetic nervous system stimulation of alpha-2 receptors in the smooth muscle surrounding the digital arteries that respond to norepinephrine [8]. Endothelial hypertrophy is assumed to reduce the arterial width and put these arteries at risk for ischemia in the event of a vasospasm [9].

Raynaud symptoms, when linked to an underlying disease like systemic sclerosis, scleroderma, Sjögren, or mixed connective tissue disease, are referred to as Raynaud phenomenon or secondary RP [5,8]. Within this context, RP manifestations are believed to arise as a result of the growth of fibrous connective tissue and intimal lesions in medium-sized arteries [9].

The off-label utilization of botulinum toxin A for the management of arterial vasospasm was first observed in 2003 [10]. In 2004, Sycha et al. [8] published the initial report in the literature that showcased the effectiveness of this treatment for retinitis pigmentosa (RP). The study involved two patients, one with primary RP and the other with secondary RP. Following the treatment, both patients exhibited enhancements in subjective symptoms and laser Doppler imaging flow. Subsequently, there have been other studies in the literature documenting the effective decrease in RP symptoms following the administration of botulinum toxin A. A cumulative dose of 70E was administered to both hands of the patient, with 35E distributed to each of the specified injection sites. Botulinum toxin type A was injected at the base of the fingers. The patient's symptoms showed improvement on the third day, marked by increased hand temperature, lack of swelling and pain, and a lasting effect lasting for three months. Therefore, our findings align with the prior studies mentioned.

While botulinum toxin A is not currently licensed by the FDA for treating RP, it is believed to be an effective therapeutic method due to many causes. The interaction between the toxin and skeletal muscle entails the binding of SNAP-25 (synaptosomal-associated protein, 25kDa) proteins, which are responsible for the transportation and release of acetylcholine vesicles at the presynaptic end of the neuromuscular junction. This binding leads to a transient state of muscular paralysis [11]. Paralysis commonly manifests within a timeframe of one to four days following exposure, persisting for a duration of two to four months. This timeframe is deemed sufficient for the resynthesis of inactivated SNAP-25 proteins [1]. The participation of this process in treating RP symptoms is believed to be minor, as many of the therapeutic effects have been shown to persist beyond this specific timeframe [1]. The symptoms exhibited by our patient demonstrated improvement on the third day, characterized by elevated hand temperature, absence of swelling and pain, and a sustained impact spanning a duration of three months. Consequently, we establish a correlation with the findings reported by Serri et al. [11].

Regarding the mechanism underlying chemical sympathectomy, it has been postulated that botulinum toxin A functions by inhibiting the recruitment of alpha-2 receptors, which are known to induce vasoconstriction, to the smooth muscle of blood vessels [10]. A number of scholars have proposed that the toxin could potentially impede the subsequent release of pain-related chemical mediators, including glutamate, calcitonin gene-related peptide, and substance P [10,11]. Our patient did not exhibit any pain or swelling on the hands following the therapy, establishing a correlation between our findings and the aforementioned studies. The application of botulin toxin is commonly indicated in several medical conditions including focal dystonia, spasticity, paraparesis in children with cerebral palsy, post-stroke conditions, multiple sclerosis, brain injuries, involuntary muscle hyperactivity of a non-dystonic nature, pain management, strabismus, nystagmus, sialorrhea, and esthetic medicine [2-4].

Contraindications include anyone with proven allergies or sensitivities to the toxin, pregnant patients, lactating moms, and those with a persistent infection. Neuromuscular diseases, such as myasthenia gravis, are considered to be relative contraindications. The toxin should not be administered to patients who are currently on treatment by drugs that reduce neuromuscular transmission [4,5]. The drugs included in this category consist of calcium channel blockers, penicillamine, aminoglycosides, lincosamides, polymyxins, magnesium sulfate, anticholinesterases, succinylcholine, and quinidine [6].

In 2004, a pilot study was conducted by Sycha et al., which involved two patients diagnosed with severe Raynaud's phenomenon [8]. These patients had an outstanding response to infiltration with botulinum toxin. Similar findings have also been documented in prior research [10-13]. Van Beek et al. [7] conducted a study including 11 patients and observed a reduction in pain within the initial 24-48 hours. Additionally, they noted a decrease in the occurrence of vasospasm episodes, which persisted for 9.6 months throughout the follow-up period. The ulcers in nine patients have undergone healing. The experienced pain showed a reduction from 9-10 to 0-2 [10]. In our case, the patient's symptoms showed improvement on the third day, marked by increased hand temperature, lack of swelling and pain, and a lasting effect lasting for three months. Therefore, our findings align with Sycha et al. [8].

In 2009, Neumeister [2] conducted a study involving 19 patients who received an injection of 50 to 100 IU of botulinum toxin type A per hand. The study found that 10 out of the 14 patients experienced an 84% decrease in pain and improved blood flow in the Doppler study 30 minutes after the injection. Additionally, all patients experienced ulcer healing within 60 days. However, Bello et al. [10] conducted a recent study, wherein they were unable to establish any enhancement in flow during the Doppler examination following the administration of botulinum toxin to individuals diagnosed with Raynaud's phenomenon resulting from systemic scleroderma [11]. Our patient exhibited improvement on the third day, characterized by increased temperature in both hands and enhanced blood circulation after the injection. These findings align with the findings of Neumeister [2].

The study conducted by Serri et al. [11] revealed that a group of 18 individuals diagnosed with scleroderma and Raynaud's phenomenon experienced notable improvements in pain levels as assessed by the QuickDASH scale, as well as enhanced partial pressure of oxygen and complete healing of ulcers [12]. In a prospective trial conducted by Motegi et al. [12], a group of 10 Japanese patients diagnosed with scleroderma and Raynaud's phenomenon exhibited a reduction in the occurrence of episodes, along with enhancements in color, length, and pain levels. Additionally, the authors observed the cure for digital ulcers. Furthermore, thermography was employed to quantify alterations in finger temperature after submersion in cold water [13]. In a recent retrospective study by Medina et al. [13] which included 15 patients with Raynaud’s syndrome, they reported that 30 minutes after the infiltration of the toxin, six patients already showed a response, and four of them were very good responders. One of the two proposals by Fregene et al. [9] involves using an infiltration technique at the base of the fingers of patients. These findings indicate that there are no notable variations in effectiveness, although there is a higher likelihood of weakness in the intrinsic musculature when the injection is administered closer to the palm or wrist [9].

A notable improvement in the patient's symptomatology was observed on the third day, characterized by the occurrence of hand warming without any accompanying swelling or discomfort. This improvement persisted for a length of three months. A six-month follow-up was conducted on the patient subsequent to the administration of botulinum toxin type A therapy, during which no signs of recurrence or progression of RS were observed. By the end of the sixth month, there was a recurrence of symptoms characterized by changes in color, sensations of pain, and increased sensitivity on the outside side of the index finger. The results of our study indicate a significant association between the administration of botulinum toxin type A and the therapeutic outcomes, which aligns with the findings reported by Fregene et al. [9], Serri et al. [11], Motegi et al. [12], and Medina et al. [15].

Adverse effects associated with botulinum toxin injections are rare [10]. The injection location may experience local side effects such as discomfort, ecchymosis, or intrinsic muscle weakening. Endogenous paralysis is transient and has a duration of two to four months. Systemic adverse effects that are infrequent are temporary and may encompass symptoms like those of influenza, such as nausea, fatigue, malaise, headache, runny nose, diplopia, dysarthria, generalized muscle weakness, asthenia, blurred vision, ptosis, dysphagia, dystonia, urine incontinence, breathing difficulties, and rashes [8-10]. In the current case, no complications were noted in the patient following the treatment. No indications of swelling or pain were observed.

Fregene et al. [9] suggested performing clinical follow-up after one, three, and six months following botulinum toxin A treatment and as necessary for further treatment in the case of potential recurrence of RS. In the present case, the patient underwent a six-month follow-up after receiving botulinum toxin type A therapy, during which no indications of recurrence or advancement of RS were detected. Following the conclusion of the sixth month, a reoccurrence of symptoms was observed, which manifested as changes in color, perceptions of pain, and increased sensation on the exterior side of the index finger.

## Conclusions

In conclusion, this case report demonstrates the effective utilization of botulinum toxin type A, for the treatment of Raynaud's syndrome in a female patient aged 70 years. Significant improvements in symptomatology were observed through the implementation of a comprehensive administration approach and ongoing monitoring of treatment outcomes. These benefits included pain reduction, increased hand warmth, and the absence of swelling and pain for a duration exceeding three months.

The lack of negative complications highlights the safety characteristics of botulinum toxin A within this specific case. The results mentioned above add to the expanding collection of information that substantiates the effectiveness of botulinum toxin A as a viable treatment choice for Raynaud's syndrome. This underscores its ability to improve the well-being of patients and justifies the need for research in more extensive clinical trials.
